# Case Report: Identification of a novel pathogenic *UGT1A1* mutation in a Chinese patient with Gilbert syndrome

**DOI:** 10.3389/fmed.2025.1581923

**Published:** 2025-10-15

**Authors:** Chenyu Zhao, Hui Huang

**Affiliations:** ^1^Department of Gastroenterology, Henan Provincial People’s Hospital, People’s Hospital of Zhengzhou University, Zhengzhou, China; ^2^Department of Medical Genetics, Hunan Province Clinical Research Center for Genetic Birth Defects and Rare Diseases, The Second Xiangya Hospital, Central South University, Changsha, China

**Keywords:** Gilbert syndrome, *UGT1A1*, mutation, hyperbilirubinemia, pathogenic

## Abstract

Gilbert syndrome (GS) is a genetic disorder caused by mutations in the *UGT1A1* gene. It is characterized by intermittent non-hemolytic unconjugated hyperbilirubinemia. Herein, we report a patient with GS who presented with chronic hyperbilirubinemia and no other abnormal manifestations. Heterozygous c.1047_1047delG, c.-3279 T>G, and c.-41_-40dupTA mutations were identified in his *UGT1A1* gene by using Sanger sequencing. The novel c.1047_1047delG variant was classified as a pathogenic mutation. These findings not only provide a basis for the genetic diagnosis of this GS patient but also expand the variant database of the *UGT1A1* gene.

## 1 Introduction

Gilbert syndrome (GS; OMIM #143500) is an autosomal recessive genetic disorder characterized by intermittent unconjugated hyperbilirubinemia in the absence of hemolysis, with no other features of hepatobiliary dysfunction (1). First described in 1901 by Gilbert and Lereboulet (2), its population incidence has been reported to be approximately 3–13% (3, 4). The *UGT1A1* gene (OMIM 191740) encodes uridine diphospho-glucuronosyltransferase 1A1 (UGT1A1), an enzyme primarily localized to the smooth endoplasmic reticulum of hepatocytes. Pathogenic variants in the *UGT1A1* gene are the underlying cause of GS. Loss-of-function mutations in this gene reduce UGT1A1 enzyme activity, thereby impairing the conjugation of bilirubin with glucuronic acid. Importantly, GS does not impact life expectancy and typically requires no specific treatment. To date, the Human Gene Mutation Database (HGMD^1^) has cataloged 189 mutations in the *UGT1A1* gene. In the present study, we report a patient diagnosed with GS and identify novel pathogenic variant in the *UGT1A1* gene (NM_000463.3): c.1047_1047delG, g.7652_7652delG, and p.I350Yfs16.

## 2 Case report and genetic testing

**Figure 1:**
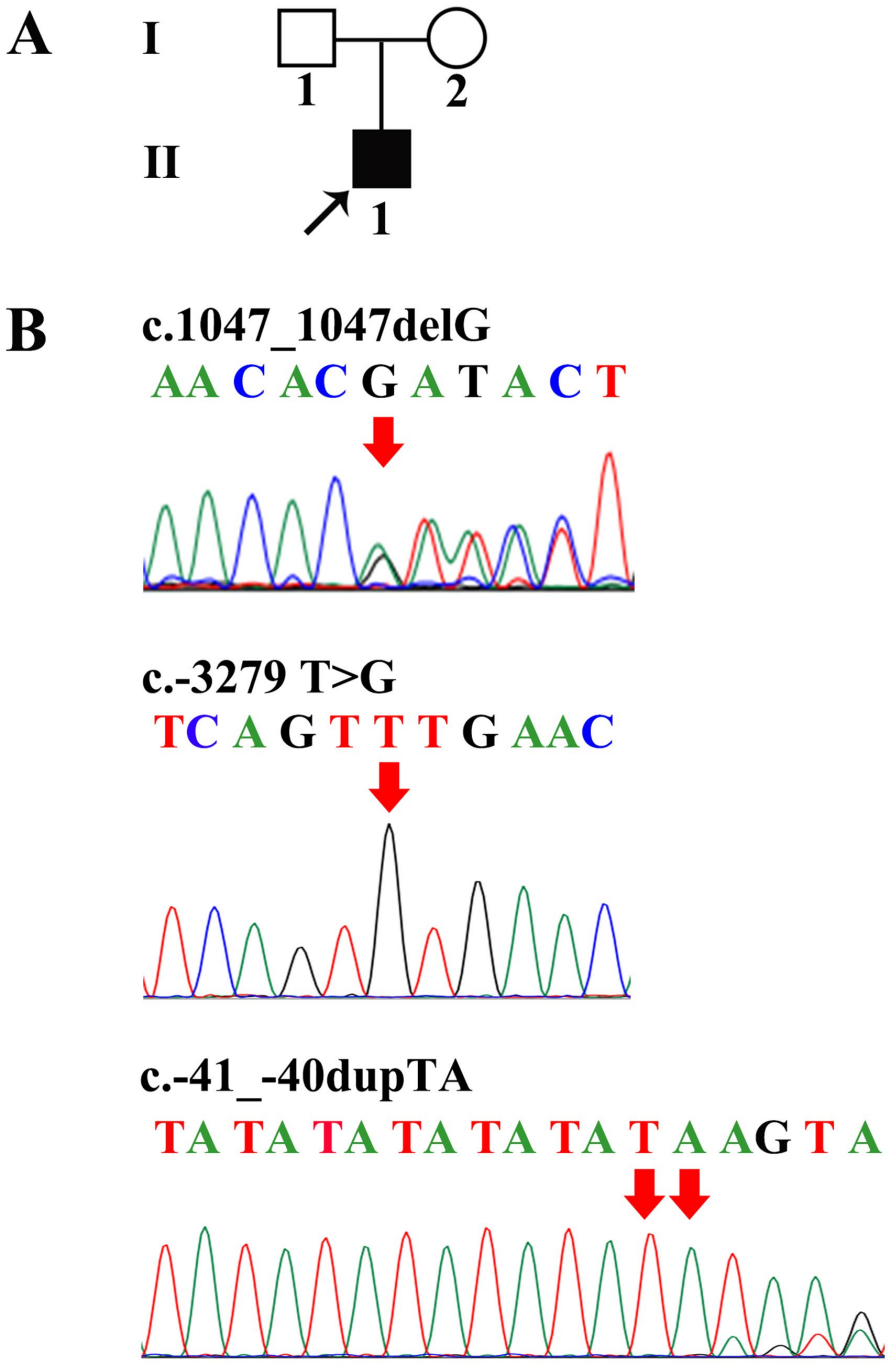
Family tree and *UGT1A1* genetic analysis. **(A)** Proband II-1 suffered from GS. **(B)** Direct sequencing showed heterozygous c.1047_1047delG, c.-3279 T>G, and c.-41_-40dupTA mutations in II-1.

A 17-year-old man had experienced intermittent mild jaundice for more than 8 years. The jaundice was occasionally accompanied by dull pain in the right upper quadrant and was notably triggered by fatigue and fasting. It resolved spontaneously within 1–2 weeks. Throughout the disease course, there was no pruritus, darkening of urine, or clay-colored stools. He had no prior medical history, nor any remarkable personal or family history (Figure 1A). At the time of presentation to our hospital, he exhibited yellowing of the skin and sclera. Laboratory tests revealed a total bilirubin (TBIL) level of 74.2 μmol/L (normal range: 3.4–17.1 μmol/L) and a direct bilirubin (DBIL) level of 68.4 μmol/L (normal range: 0–6 μmol/L); no other significant abnormalities were noted in his liver function tests. Hemolytic tests yielded negative results, and he was also negative for hepatitis B virus (HBV) infection. Ultrasonographic examination of the liver, biliary tract, pancreas, and spleen showed no abnormalities.

Genomic DNA was isolated from the peripheral blood leukocytes of the patient. The promoter region (encompassing the TATA box), enhancer region [Phenobarbital-responsive enhancer module (PBREM)], coding exons, and splice junctions of the *UGT1A1* gene were amplified via polymerase chain reaction (PCR). The primer sequences used are provided in Table 1. PCR products were subjected to Sanger sequencing, and variants in *UGT1A1* were identified by aligning the sequencing results with the published reference sequence (GenBank accession no.: NM_000463). The pathogenicity of the identified mutations was interpreted in accordance with the guidelines of the American College of Medical Genetics and Genomics (ACMG) (5). The patient provided written informed consent for participation in this study. However, his parents declined to donate their blood samples. This study was approved by the Ethics Committee of the Second Xiangya Hospital of Central South University. Subsequent genetic analysis revealed that the patient carried three heterozygous mutations in *UGT1A1*: c.1047_1047delG, c.-3279T>G, and c.-41_-40dupTA (corresponding to the UGT1A1*28 allele) (Figure 1B). During telephone follow-up, the patient still reported intermittent jaundice.

**Table 1:** Primers sequences of the *UGT1A1* gene.

*UGT1A1* exon	Primer sequence (5′ → 3′)
Exon 1–1	F: TATAAGTAGGAGAGGGCGAACC
	R: TCAAATTCCAGGCTGCATG
Exon 1–2	F: GGCCTCCCTGGCAGAAAG
	R: ATGCCAAAGACAGACTCAAACC
Exon 2	F: AGGAACCCTTCCTCCTTTAGA
	R: GAAGCTGGAAGTCTGGGATTAG
Exon 3	F: CCTCAGAAGCCTTCACAGTTAC
	R: ATCCAATCCGCCCAACATAC
Exon 4	F: GTGTCCAGCTGTGAAACTCA
	R: TGAATGCCATGACCAAAGTATTC
Exon 5	F: CAACAGGGCAAGACTCTGTATC
	R: CCTTATTTCCCACCCACTTCTC
Promoter	F: ACAGGTTTCCATGGCGAAAG
	R: TGTTTTGATCACACGCTGCA
PBREM	F: GGTCACTCAATTCCAAGGGG
	R: GCATCCAAGCCAGCAAGTAA

PBREM, phenobarbital responsive enhancer module; UGT1A1, uridine diphosphate glucuronosyltransferase 1A1; F, forward; R, reverse; bp, base pairs.

## 3 Discussion

The UGT1A1 enzyme is predominantly expressed in the liver, where it localizes to the membrane of the smooth endoplasmic reticulum. Its primary function is to catalyze the conjugation of bilirubin with glucuronic acid. Variations in the *UGT1A1* gene can lead to varying degrees of quantitative reduction in UGT1A1 enzyme activity, thereby causing inherited non-hemolytic unconjugated hyperbilirubinemia (UCH). The classification of Crigler–Najjar syndrome type I (CNS-I, OMIM#218800), Crigler–Najjar syndrome type II (CNS-II, OMIM#606785), and GS subtypes largely relies on serum bilirubin levels (6). However, these conditions are now recognized as representing a continuous clinical spectrum of a single underlying disorder. Specifically, UGT1A1 activity is reduced to approximately 30, 10, and 0% of normal levels in patients with GS, CNS-II, and CNS-I, respectively. Notably, some cases of neonatal hyperbilirubinemia and breast milk jaundice may be classified as transient familial neonatal hyperbilirubinemia (OMIM#237900), a condition associated with UGT1A1 gene polymorphisms or mutations (7). The patient’s TBIL levels fall within the range for GS (<100 μmol/L), and the predominance of unconjugated bilirubin is consistent with the pathophysiology of GS. In contrast, CNS-II is characterized by more severe UCH. In CNS-II, TBIL levels are usually 100–400 μmol/L. Additionally, the patient had no history of neonatal jaundice requiring prolonged phototherapy—another key clinical differentiator of CNS-II, which often presents with severe neonatal hyperbilirubinemia as a key feature.

**Figure 2:**
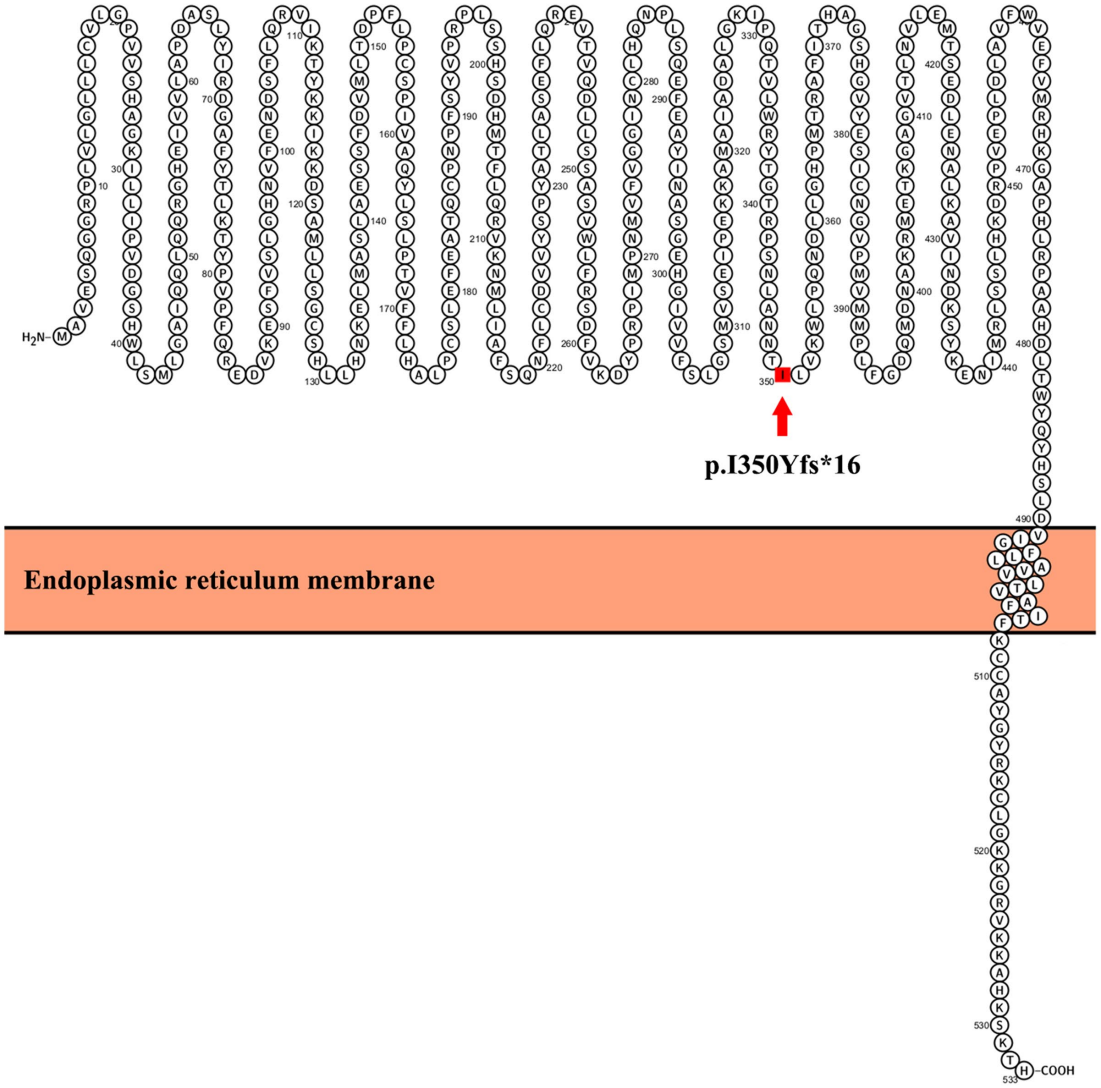
Location of the mutation in UGT1A1. The schematic diagram of UGT1A1 was generated using Protter software (http://wlab.ethz.ch/protter/start/).

The UGT1A1 protein contains several key structural domains that are critical for its enzymatic function in glucuronidation reactions. These domains include: N-terminal substrate-binding domain, C-terminal UDP-glucuronic acid (UDPGA) binding domain, interdomain linker region, transmembrane domain, and cytoplasmic tail. The p.I350Yfs*16 variant in *UGT1A1* is a frameshift mutation that introduces a premature termination codon 16 amino acids after the frameshift at position 350. This mutation significantly impacts the protein structure by truncating the C-terminal region, with key effects on critical functional domains. The location of this mutation within UGT1A1 is shown in Figure 2. The variant was evaluated using two widely recognized in silico tools, MutationTaster (8) and PROVEAN (9), both of which consistently predicted pathogenic effects.

The p.I350Yfs*16 variant is a frameshift mutation that disrupts the open reading frame of the *UGT1A1* gene, which directly aligns with the “null variant” definition for very strong pathogenic evidence (PVS1). This variant was not detected in large population databases, including the 1000 Genomes Project (1000G), the Exome Aggregation Consortium (ExAC), and the Genome Aggregation Database (gnomAD) (Pathogenic Moderate evidence 2, PM2). Prediction software, MutationTaster and PROVEAN, predicted a deleterious effect of the variant (Pathogenic Supporting evidence 3, PP3). Per ACMG’s variant classification framework, the combination of 1 very strong criterion (PVS1) + 1 moderate criterion (PM2) + 1 supporting criterion (PP3) meets the threshold for pathogenic classification. Additionally, a comprehensive search of the HGMD and a review of previously published literature confirmed that this *UGT1A1* variant has not been reported before, thus identifying it as a novel mutation.

Two other known mutations are also associated with GS. The first is a TA insertion in the TATA box of the *UGT1A1* promoter, resulting in the [A(TA)₇TAA] genotype. This variant reduces *UGT1A1* gene expression by 50–70% (10–12). The second is the c.-3279 T>G variant located in the PBREM, which leads to an approximately 40% decrease in UGT1A1 enzyme activity (13).

The present study is subject to several limitations. The parental sequencing would have further clarified variant inheritance, but it was not feasible due to parental refusal. In addition, we did not perform *in vitro* functional validation to experimentally confirm the functional impact of the novel c.1047_1047delG variant. However, these limitations do not affect the interpretation of the variant’s pathogenicity, as the variant meets ACMG pathogenic criteria.

## 4 Conclusion

In conclusion, this study identifies a novel pathogenic mutation in the *UGT1A1* gene. These findings not only facilitate the genetic diagnosis of families affected by GS but also expand the known spectrum of *UGT1A1* mutations.

## Data Availability

The datasets for this article are not publicly available due to concerns regarding participant/patient anonymity. Requests to access the datasets should be directed to the corresponding author.

## Footnotes

^1^ http://www.hgmd.cf.ac.uk/ac/index.php
